# Biscuit consumption and diabetic retinopathy incidence in adults in the United States

**DOI:** 10.1186/s13098-022-00860-7

**Published:** 2022-07-06

**Authors:** Ke Shi, Yuhong Chen, Xinyue Zhu, Jiali Wu, Jieqiong Chen, Jing Hu, Xiaodong Sun, Jingfa Zhang

**Affiliations:** 1 Department of Ophthalmology, Shanghai General Hospital, Shanghai Jiao Tong University School of Medicine, 100 Hai Ning Road, 200080 Shanghai, People’s Republic of China; 2 National Clinical Research Center for Eye Diseases, Shanghai, China; 3 Shanghai Key Laboratory of Fundus Diseases, Shanghai, China; 4 Shanghai Engineering Center for Visual Science and Photomedicine, Shanghai, China

**Keywords:** Dietary, Biscuit, Diabetic retinopathy, The National Health and Nutrition Examination Survey, Binary logistic regression model.

## Abstract

**Background:**

Foods have a considerable influence on human health and were directly related to glycemic control for diabetes patients. However, little is known about the effects of biscuits, a traditional food consumed in large amounts in several countries, on diabetic retinopathy. This study aimed to explore the association between biscuit consumption and diabetic retinopathy prevalence in adults of the United States population.

**Methods:**

A cross-sectional study with 1904 participants from the National Health and Nutrition Examination Survey database were included in this population-based, cross-sectional study. The association between different consumption frequencies of biscuit and diabetic retinopathy prevalence was evaluated using a binary logistic regression model. Trend test, stratified and interaction analyses were also performed.

**Results:**

After possible confounders including sex, age, ethnicity, education, marital status, family poverty income ratio, smoking and alcohol consumption habit, fasting blood glucose level, hemoglobin A1c level, diagnosis of diabetes, insulin use, blood pressure, body mass index were adjusted, the participants who consumed biscuit 1–11 times a year, 1–3 times a month, and more than once a week had a 139.8% (95% confidence interval, 1.003–5.734), 182.1% (95% confidence interval, 1.106–7.191), and 236.2% (95% confidence interval, 1.335–9.844) higher risk of diabetic retinopathy prevalence, respectively, compared with those who never ate biscuit. For male, non-Hispanic, and overweight (body mass index ≥ 25 kg/m^2^) subgroups, the trend test demonstrated that the diabetic retinopathy prevalence significantly elevated with increased frequency of biscuit consumption (*P*_*trend*_ = 0.021, 0.009, and 0.002, respectively). The interaction analysis suggested that no aforementioned confounders played an interactive role in the relationship between biscuit consumption and diabetic retinopathy prevalence.

**Conclusions:**

The risk of diabetic retinopathy was positively associated with biscuit consumption. Moreover, for male, non-Hispanic, or overweight individuals, the risk of diabetic retinopathy significantly increased with the frequency of biscuit consumption.

**Supplementary Information:**

The online version contains supplementary material available at 10.1186/s13098-022-00860-7.

## Background

Diabetic retinopathy (DR) is a common microvascular complication of diabetes mellitus (DM) that causes irreversible retinal microvasculopathy and neurodegeneration [1]. It remains the leading cause of vision impairment in working-aged people worldwide [2]. According to a meta-analysis, the number of adults with DR was predicted to increase from 103.12 million in 2020 to 160.50 million in 2045 [3]. Therefore, DR imposes an enormous socioeconomic burden on the global healthcare system. DR is mainly caused by hyperglycemia, and the maintenance of glycemic control is the goal of all patients with DM. According to the Diabetes Prevention Program, adopting a healthy lifestyle is beneficial to prevent or delay the onset of DM and DR in patients with DM [4].

A healthy dietary pattern is the cornerstone of DM management since diet can affect human health and is directly related to glycemic control. An unhealthy diet may play a critical role in the development of several diseases, including obesity, cardiovascular disease (CVD), cancer, and DM [5, 6]. Several studies have demonstrated that intake of diet with trans fatty acids (TFA) [7], baked goods [8], red meat [9], and fried foods [10], in addition to sweets, were positively associated with DM prevalence. Biscuits have become a traditional food consumed in large amounts in several countries [11]. The Malmö Diet and Cancer cohort study indicated that higher intake of biscuits was positively related with increased risk of non-aggressive prostate cancer [12]. To the best of our knowledge, no observational studies have investigated the relationship between biscuit consumption and DR in the United States population.

In epidemiological studies, population-level dietary exposures are assessed through food frequency questionnaires (FFQs), which are principally used to estimate long-term average intakes [13] and are well known as an effective dietary evaluation tool [14]. The National Health and Nutrition Examination Survey (NHANES) is an ongoing survey that focuses on a range of health and nutrition evaluations of the residents in the United States. Logistic regression is a classification algorithm used to predict a binary outcome based on a set of independent variables. The odds ratio, the coefficients of logistic regression, could indicate the constant effect of a predictor on the likelihood that one outcome will occur. Herein, we utilized the FFQ data from the NHANES database and performed logistic regression modeling to explore the correlation between different frequency of biscuit consumption and the risk of DR, providing dietary advice to DM patients with or without DR.

## Methods

### Data sources

This study analyzed data from the NHANES 2005–2006 cycle since it is the only available two-year cycle that contains both the raw FFQ and retinal examination profiles. All NHANES data collection protocols were approved by the ethics review board of the National Center for Health Statistics Research (https://www.cdc.gov/nchs/nhanes/irba98.htm) and all participants provided written informed consent. This cross-sectional study analyzed de-identified, free-assessed public online data (https://wwwn.cdc.gov/nchs/nhanes/) and was exempt from the approval of the ethics review board of Shanghai Jiaotong University.

### Patient and public involvement

Patients or the public were not involved in the design, or conduct, or reporting, or dissemination plans of our research.

### Exposures and confounders

The frequency of biscuit consumption, which is the main exposure in our study, was retrieved from the NHANES FFQ, a semiquantitative questionnaire listing more than 130 food items to assess dietary intake over the past year. The frequency of biscuit consumption was reclassified into four groups: never ate, 1–11 times a year, 1–3 times a month, and more than once a week. Moreover, the consumption of 24 foods (soft drinks, ham, popcorn, melons, sushi, pineapple, crackers. bananas, fruit juice, pancakes, hot dogs, cookies, cake, doughnuts, pizza, sweet muffins, cheese, pie, ice cream, beer, French fries, potato chips, chocolate candy, and chili) with glycemic index greater than 50 were identified as confounders.

Information on demographics, smoking and alcohol consumption habits, blood pressure, blood fasting glucose level, hemoglobin A1c (HbA1c) level, diagnosis of diabetes, insulin use, and body mass index (BMI) was also extracted and used as confounders. Marital status was categorized as partnered for married or living with a partner and single for unmarried, divorced, widowed, or separated. Family poverty income ratio (PIR) was calculated as the ratio of family income to the federal poverty level and classified into three groups (< 1.3, 1.3–3.5, and > 3.5) according to previous literature [15]. Blood pressure was expressed as the average of multiple consecutive measurements. BMI was classified into four groups defined by the World Health Organization: <18.5, 18.5–24.9, 25–29.9, and ≥ 30 kg/m^2^.

### Outcome

The outcome was the presence or absence of any DR, and DR assessment information of participants was obtained in the retinal imaging subsection of the NHANES ophthalmology component tests. Two non-dilated retinal digital pictures of participants aged ≥ 40 years were acquired using Canon digital cameras (CR6-45 NM and EOS-10D, Canon USA, One Canon Park, Melville, NY). Fundus photographs were assessed using the NHANES digital grading protocol at the University of Wisconsin by at least two trained graders using the EyeQ Lite software (EyeQ Inc., Calgary, Canada). Any disagreement between the first two graders on the pathological evaluation was resolved by a third grader. An adjudicator will make a final determination if two of the three graders disagree.

### Statistical analysis

All analyses were calculated accounting for the NHANES sample weights. Multigroup comparisons were performed using the Kruskal–Wallis test for continuous variables, and Fisher’s precision probability test was used for categorical variables. Binary logistic regression analyses were used to determine the potential association between biscuit consumption and DR risk in both the unadjusted and adjusted models. Models were adjusted as follows: model 1 was unadjusted, model 2 was adjusted for sex, age, and ethnicity; and model 3 was adjusted for sex, age, ethnicity, education level, marital status, family PIR, smoking and alcohol consumption habits, fasting blood glucose level, blood pressure, and BMI.

Additionally, trends over different consumption frequencies were compared using the Cochran–Armitage trend test. Missing values for fasting blood glucose levels were coded as dummy variables. No imputation was performed for other confounders, including education level, marital status, family PIR, alcohol consumption, blood pressure, and BMI because the percentage of missing data was minimal (< 4.5%). Stratified analyses were performed using the aforementioned confounders. Furthermore, we conducted a log-likelihood ratio test to describe the significant interactions between these subgroups. All statistical analyses were performed using the R software (version 4.0.4; R Foundation for Statistical Computing, Vienna, Austria) and EmpowerStats (version 2.0; X&Y Solutions Inc., Boston, MA, USA). A *P*-value < 0.05 was considered significant.

## Results

### Characteristics of participants

**Fig. 1:**
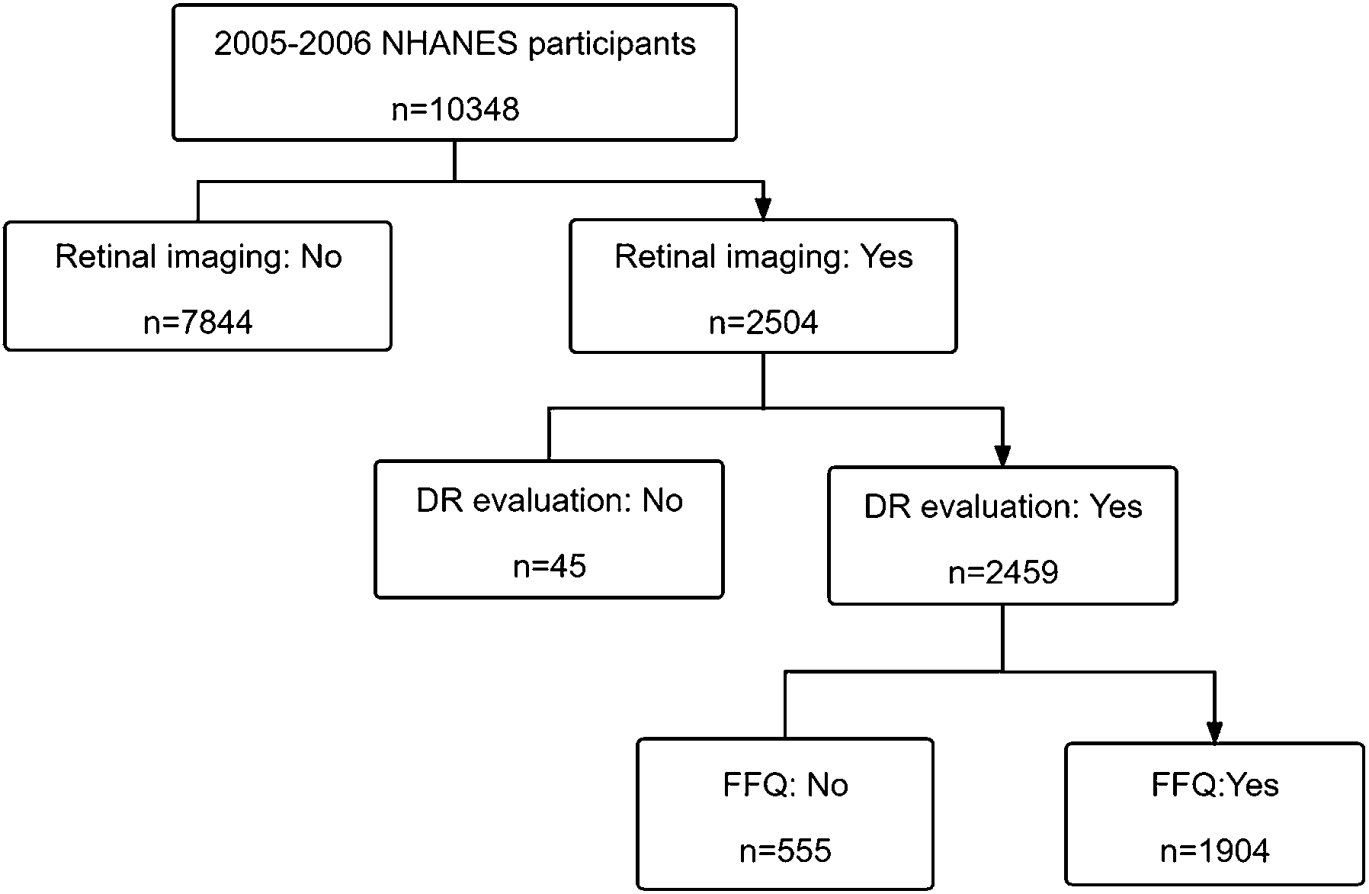
Flowchart of study procedures showing participant selection. The schematic illustrates the participants included and excluded for the present study from the 2005–2006 NHANES database. NHANES, National Health and Nutrition Examination Survey; FFQ, food frequency questionnaires; DR, diabetic retinopathy

The flowchart depicting the study inclusion and exclusion is shown in Fig. 1. Altogether, 10,348 individuals participated in the NHANES 2005–2006 cycle, and 7844 participants who had no retinal data were excluded. Moreover, among the remaining 2504 participants, 45 without DR evaluation and 555 without FFQ information were subsequently excluded. Finally, 1904 participants with valid DR evaluation and available food intake frequency information were included in this study.

Table 1 presents the characteristics of participants according to DR status. Participants with DR were significantly more likely to be male (*P* = 0.047) and elder (*P* < 0.001) and have a lower PIR (*P* < 0.001), higher systolic blood pressure (*P* < 0.001), and higher BMI (*P* = 0.005).

**Table 1:** Descriptive characteristics of the 1904 participants stratified by DR status

	No DR (n = 1657)	Any DR (n = 247)	*P*-value
**Sex**			**0.047**
Male	820 (49.49%)	139 (56.28%)	
Female	837 (50.51%)	108 (43.72%)	
**Age (years)**	59.70 ± 12.91	63.32 ± 12.39	** < 0.001**
**Ethnicity**			** < 0.001**
Mexican American	233 (14.06%)	42 (17.00%)	
Other Hispanic	32 (1.93%)	6 (2.43%)	
Non-Hispanic White	1037 (62.58%)	117 (47.37%)	
Non-Hispanic Black	303 (18.29%)	75 (30.36%)	
Other ethnicities	52 (3.14%)	7 (2.83%)	
**Education**			** < 0.001**
Lower than 9th grade	168 (10.14%)	39 (15.79%)	
9–11th grade (includes 12th grade with no diploma)	230 (13.88%)	48 (19.43%)	
High school grade/General education diploma or equivalent	431 (26.01%)	66 (26.72%)	
Some college or associate of arts degree	441 (26.61%)	67 (27.13%)	
College graduate or above	386 (23.30%)	27 (10.93%)	
Not recorded	1 (0.06%)	0 (0.00%)	
**Marital status**			0.861
Partnered	1073 (64.76%)	160 (64.78%)	
Single	582 (35.12%)	87 (35.22%)	
Not recorded	2 (0.12%)	0 (0.00%)	
**Family PIR**	2.93 ± 1.59	2.52 ± 1.45	** < 0.001**
**Had at least 12 alcohol drinks in one year**			**0.015**
Yes	1144 (69.04%)	149 (60.32%)	
No	487 (29.39%)	95 (38.46%)	
Not recorded	26 (1.57%)	3 (1.21%)	
**Smoked at least 100 cigarettes in life**			0.663
Yes	910 (54.92%)	132 (53.44%)	
No	747 (45.08%)	115 (46.56%)	
**Fasting blood glucose level (mmol/l)**	5.92 ± 1.47	7.22 ± 3.01	** < 0.001**
**Hemoglobin A1c (HbA1c, %)**	5.61 ± 0.77	6.58 ± 1.68	** < 0.001**
**Diagnosis of diabetes**			** < 0.001**
Yes	153 (9.23%)	104 (42.11%)	
No	1464 (88.35%)	136 (55.06%)	
Borderline	40 (2.41%)	6 (2.43%)	
Not recorded	0 (0.00%)	1 (0.40%)	
**Insulin use**			** < 0.001**
Yes	25 (1.51%)	52 (21.05%)	
No	1632 (98.49%)	195 (78.95%)	
**Systolic blood pressure (mmHg)**	128.14 ± 19.51	136.75 ± 23.44	** < 0.001**
**Diastolic blood pressure (mmHg)**	70.88 ± 13.74	71.04 ± 14.50	0.867
**BMI (kg/m** ^**2)**^	29.04 ± 6.79	30.36 ± 6.90	**0.005**
**Biscuit consumption frequency**			** < 0.001**
Never ate	234 (14.12%)	17 (6.88%)	
1–11 times per year	824 (49.73%)	119 (48.18%)	
1–3 times per month	386 (23.30%)	60 (24.29%)	
≥ 1 times per week	187 (11.29%)	42 (17.00%)	
Not recorded	26 (1.57%)	9 (3.64%)	

Mean ± SD for continuous variables.

Boldface indicates statistical significance.

PIR, poverty income ratio; BMI, body mass index; DR, diabetic retinopathy.

### Relationship between DR prevalence and biscuit consumption

As shown in Table 2, univariate analysis was performed to identify the relationship between the aforementioned covariates and DR prevalence. We found that female (*P* = 0.047), age ≥ 60 years (*P* < 0.001), non-Hispanic White ethnicity (*P* = 0.016), less alcohol drinking (*P* = 0.004), higher family PIR (*P* = 0.007), and systolic blood pressure ≥ 120 mmHg (*P* < 0.001) were all significantly associated with DR.

**Table 2:** Univariable analysis of the effects of covariates on DR

	n (%)	OR(95% CI)	*P*-value	*P*_trend_
**Sex**				
Male	959 (50.37%)	Reference		**0.047**
Female	945 (49.63%)	0.761 (0.582, 0.996)	**0.047**	
**Age**				
< 50	498 (26.16%)	Reference		** < 0.001**
50–60	418 (21.95%)	1.446 (0.932, 2.242)	0.099	
60–70	477 (25.05%)	2.179 (1.459, 3.255)	** < 0.001**	
≥ 70	511 (26.84%)	2.069 (1.389, 3.083)	** < 0.001**	
**Ethnicity**				
Mexican American	275 (14.44%)	Reference		0.436
Other Hispanic	38 (2.00%)	1.040 (0.410, 2.641)	0.934	
Non-Hispanic white	1154 (60.61%)	0.626 (0.428, 0.915)	**0.016**	
Non-Hispanic black	378 (19.85%)	1.373 (0.907, 2.079)	0.134	
Other ethnicities (including multi-racial)	59 (3.10%)	0.747 (0.318, 1.756)	0.503	
**Education level**				
Less than 9th grade	207 (10.87%)	Reference		** < 0.001**
9–11th grade (includes 12th grade with no diploma)	278 (14.60%)	0.899 (0.564, 1.434)	0.655	
High school grade / General education diploma or equivalent	497 (26.10%)	0.660 (0.427, 1.018)	0.060	
Some college or associate of arts degree	508 (26.68%)	0.654 (0.424, 1.009)	0.055	
College graduate or above	414 (21.74%)	0.301 (0.178, 0.507)	** < 0.001**	
**Marital status**				
Partnered	1233 (64.83%)	Reference		0.986
Single	669 (35.17%)	1.002 (0.758, 1.326)	0.986	
**Family PIR**				
< 1.3	401 (21.97%)	Reference		**0.004**
1.3–3.5	725 (39.73%)	0.986 (0.702, 1.386)	0.937	
≥ 3.5	699 (38.30%)	0.601 (0.415, 0.870)	**0.007**	
**Smoked at least 100 cigarettes in life**				
Yes	1042 (54.73%)	Reference		0.664
No	862 (45.27%)	1.061 (0.812, 1.388)	0.664	
**Had at least 12 alcohol drinks in one year**				
Yes	1293 (68.96%)	Reference		**0.004**
No	582 (31.04%)	1.498 (1.134, 1.978)	**0.004**	
**Blood fasting glucose level (mmol/l)**				
< 6.9	772 (85.12%)	Reference		** < 0.001**
≥ 6.9	135 (14.88%)	4.260 (2.802, 6.478)	** < 0.001**	
**Hemoglobin A1c (HbA1c, %)**				
< 6.5	1654 (88.73%)	Reference		** < 0.001**
≥ 6.5	210 (11.27%)	7.13 (5.17, 9.82)	** < 0.001**	
**Diagnosis of diabetes**				
Yes	257 (13.50%)	Reference		** < 0.001**
No	1600 (84.08%)	0.14 (0.10, 0.19)	** < 0.001**	
Borderline	46 (2.42%)	0.22 (0.09, 0.54)	** < 0.001**	
**Insulin use**				
Yes	77 (4.04%)	Reference		** < 0.001**
No	1827 (95.96%)	0.06 (0.03, 0.09)	** < 0.001**	
**Systolic blood pressure (mmHg)**				
< 120	641 (34.37%)	Reference		** < 0.001**
120–140	755 (40.48%)	1.690 (1.186, 2.408)	**0.004**	
≥ 140	469 (25.15%)	2.507 (1.735, 3.623)	** < 0.001**	
**Diastolic blood pressure (mmHg)**				
< 80	1434 (76.89%)	Reference		0.272
80–90	308 (16.52%)	0.905 (0.615, 1.330)	0.610	
≥ 90	123 (6.60%)	1.537 (0.944, 2.502)	0.084	
**BMI (kg/m** ^**2**^ **)**				
< 18.5	25 (1.32%)	Reference		**0.014**
18.5–25	488 (25.79%)	1.168 (0.267, 5.113)	0.837	
25–30	669 (35.36%)	1.950 (0.453, 8.398)	0.370	
≥ 30	710 (37.53%)	1.907 (0.443, 8.207)	0.386	

Boldface indicates statistical significance.

NA, Not applicable. PIR, poverty income ratio; BMI, body mass index; CI, confidence interval.

The results of the binary logistic regression analysis are presented in Table 3. The association between biscuit consumption and DR prevalence was consistent among the three models, suggesting a good agreement of this positive correlation and less affected by the confounders. After adjusting for all the aforementioned confounders, compared with participants who never eat biscuit, those who consumed biscuit 1–11 times a year, 1–3 times a month, and more than once a week had a 139.8% (95% confidence interval [CI], 1.003–5.734), 182.1% (95% CI, 1.106–7.191), and 236.2% (95% CI, 1.335–9.844) higher risk of DR prevalence.

**Table 3:** Association of the biscuit consumption with DR prevalence

Frequency of biscuit consumption	OR (95% CI) *P*-value		
	Model 1	Model 2	Model 3
Never ate	Reference	Reference	Reference
1**–**11 times per year	1.988 (1.172, 3.372) **0.011**	2.119 (1.241, 3.619) **0.006**	2.398 (1.003, 5.734) **0.049**
1**–**3 times per month	2.140 (1.219, 3.755) **0.008**	2.055 (1.159, 3.645) **0.014**	2.821 (1.106, 7.191) **0.003**
≥ 1 times per week	3.092 (1.705, 5.607) **< 0.001**	2.977 (1.628, 5.443) **< 0.001**	3.362 (1.335, 9.884) **0.001**
*P* for trend	** < 0.001**	**0.003**	**0.020**

Model 1: No confounders were adjusted.

Model 2: Adjusted for sex, age and ethnicity.

Model 3: Adjusted for sex, age, ethnicity, education level, marital status, family PIR, smoking and alcohol consumption habits, fasting blood glucose level, hemoglobin A1c level, diagnosis of diabetes, insulin use, blood pressure, BMI, the consumption of 24 foods (soft drinks, ham, popcorn, melons, sushi, pineapple, crackers. bananas, fruit juice, pancakes, hot dogs, cookies, cake, doughnuts, pizza, sweet muffins, cheese, pie, ice cream, beer, French fries, potato chips, chocolate candy, and chili).

Bold values indicate statistical significance.

OR, odds ratio; CI, confidence interval.

### Stratified and interaction analyses

As shown in Table 4, the association between biscuit consumption and DR prevalence in the stratified analysis was consistent with the binary logistic regression analysis in all subgroups. Interestingly, for male, non-Hispanic, and overweight (BMI ≥ 25 kg/m^2^) subgroups, the trend test indicated that the risk of DR significantly increased with increased frequency of biscuit consumption (*P*_*trend*_=0.021, 0.009, and 0.002, respectively). The interaction analysis revealed that no confounder played an interactive role in the association between biscuit consumption and DR prevalence (Additional file 1: Table S1 for full result with all confounders).

**Table 4:** Association of biscuit consumption with DR prevalence in subgroups of confounders

	OR (95% CI) *P*-value				*P*_trend_	*P*_interaction_
	Never ate	1–11 times per year	1–3 times per month	≥ 1 times per week		
Sex						
Male	Reference	1.388 (0.678, 2.845) 0.370	1.702 (0.788, 3.677) 0.176	2.434 (1.066, 5.561) **0.035**	**0.021**	0.597
Female	Reference	2.957 (1.026, 8.520) **0.045**	2.029 (0.658, 6.254) 0.218	2.577 (0.799, 8.312) 0.113	0.585	
Ethnicity						
Hispanic	Reference	1.851 (0.534, 6.410) 0.331	0.825 (0.194, 3.501) 0.794	1.382 (0.299, 6.398) 0.679	0.673	0.529
Non-Hispanic	Reference	1.811 (0.923, 3.555) 0.084	2.209 (1.084, 4.501) **0.029**	2.689 (1.262, 5.728) **0.010**	**0.009**	
BMI (kg/m^2^)						
< 25	Reference	0.937 (0.332, 2.644) 0.902	0.943 (0.291, 3.060) 0.923	0.550 (0.141, 2.151) 0.391	0.421	0.136
≥ 25	Reference	2.759 (1.295, 5.879) **0.009**	2.768 (1.244, 6.159) **0.013**	4.376 (1.898, 10.086) **0.0005**	**0.002**	

Boldface indicates statistical significance.

OR, odds ratio; CI, confidence interval; BMI, body mass index.

## Discussion

DR is a major complication of type 1 diabetes [16]. In our study, the demographic and clinical characteristics revealed that DR were significantly more likely to be male, elder, lower PIR and individuals with higher systolic blood pressure and higher BMI. Gender was considered to be a risk factor for DR. However, some considered male gender as risk factor [17] while others identified female gender as a risk factor [18]. Our result was consistent with the study by Zhang et al. [19] that male account for 50.1% of DR patients. However, Wong et al. [20] and Park et al. [21] e the prevalence of DR in male was 47.3% and 48.3%, respectively. Various studies found that the prevalence of DR increased with age due to longer exposure to hyperglycemia[18, 22, 23], which is consistent with our result. It was estimated that nearly 80% of those with diabetes live in low- and middle-income countries [24]. Hsu et al. found that poverty is related to an increase in diabetes development in an Asian population [25], which is similar with our result. Deficiencies in the management of blood glucose levels and inequality diabetes care might contribute to the vulnerability of low-income populations to DR. Hypertension and higher BMI had been widely reported as risk factors for DR [17, 26, 27], which is consistent with our result. Hypertension would cause increased retinal blood flow and lead to retinal hyperperfusion, a critical source of injury in DR associated with shearing damage to capillaries. The effect of elevated BMI on DR might be through irregulated blood viscosity, platelet function, oxidative stress and retinal inflammation[28].

[17, 26, 28]It is well known that dietary intake have a great impact on the risk of several chronic diseases, including DM and obesity [29]. Therefore, it is essential to investigate the potential correlation between food consumption and health outcomes. Regular consumption of a fruit- and vegetable-rich diet is inversely related to the risk of DM or diabetic complications [30, 31]. In contrast, consumption of red or processed meat, eggs, and sugar-sweetened beverages was positively related to the risk of DM [32, 33]. Nevertheless, to the best of our knowledge, this study is the first investigation to assess the relationship between biscuit consumption and DR. Our results suggested that biscuit consumption was positively associated with a higher risk of DR among adults in the United States. Additionally, for male, non-Hispanic, or overweight individuals, more frequent biscuit consumption resulted in a higher risk of DR.

Although biscuits are consumed worldwide, previous studies have discovered that a dietary pattern characterized by high biscuit consumption was associated with CVD, DM, and all-cause mortality [34, 35]. Papadimitriou et al. [36] have reported that biscuit consumption was associated with a higher risk of low-grade prostate cancer in the European Prospective Investigation into Cancer and Nutrition, which was also replicated in the Netherlands Cohort Study. An African cross-sectional study has proposed that the consumption of snacks such as biscuit was significantly related to overweight and obesity [37]. These findings regarding the adverse effects of biscuit consumption are consistent with our findings. Our results suggested that eating biscuit was positively associated with an increased risk of DR, irrespective of the frequency of consumption.

The negative role of biscuit may be attributed to their ingredients, saturated fatty acids (SFA), TFA and gluten [11, 38, 39]. Although there are no studies on the relationship between fatty acids and DR, SFA and TFA have been reportedly involved in DM, CVD, and cancer [7, 40]. A Chinese cohort study has reported that total SFA and even-chain SFA intake were positively related to mortality in women.[41] Mozafarinia et al. have reported that the consumption of SFA increases the risk of breast cancer in postmenopausal women. Similarly, TFA has been suggested to be related to coronary heart disease mortality and all-cause mortality [42]. Additionally, TFA were contributory factors to obesity [43], insulin resistance [44], lymphomas [45], colorectal cancer [46], and systemic chronic inflammation [47]. The underlying mechanisms of the detrimental influence of SFA and TFA on DM might be related to endoplasmic reticulum (ER) calcium release, ER stress[48], and oxidative stress [49], leading to pancreatic β-cell impairment. Although most conventional biscuits were made with low-gluten flour, the potential influence of gluten on human health has attracted attention. Gluten-related disorders represent a series of diverse clinical manifestations caused by the ingestion of gluten [50]. Coeliac disease, the best recognized amongst these gluten-related disorders, was revealed to be an independent risk factor for DR and diabetic nephropathy in patients with type 1 diabetes [51, 52]. Evidence of the interaction between ingested gluten and the subsequent development of type 1 diabetes has been reported by various studies in humans and animals. Gluten may affect diabetes development by affecting proportional alterations in immune cell populations or by regulating the cytokine/chemokine pattern towards an inflammatory profile [53]. Therefore, gluten was speculated to be an etiopathogenesis factors for development of diabetes and gluten-free diet was suggested for susceptible individuals of diabetes [54].

Moreover, acrylamide, which forms during the thermal processing of carbohydrate-rich foods, is found in 95.5% of biscuits [55]. Acrylamide has been recognized to play carcinogenic, mutagenic, neurotoxic, and endocrine disruptive roles in living organisms. Lee et al.[56] have identified that acrylamide could induce adipocyte differentiation and obesity in mice through the regulation of mitogen-activated protein kinases and the 5´ AMP-activated protein kinase–acetyl-CoA carboxylase pathway. Acrylamide treatment was also observed to cause β-cell mass reduction in rats [57], and hemoglobin adducts of acrylamide are significantly associated with DM among those aged ≥ 20 years in the United States [58].

Our study has several limitations. First, the information of the NHANES FFQ was self-reported, so it was prone to recall bias. Second, the research population was limited to the United States, and whether this conclusion applies to European, Asian and other populations remains to be studied. Third, because of the cross-sectional design of this study, the causal association between biscuit consumption and the risk of DR was not proven. Further studies exploring the relationship between biscuit consumption and DR prevalence in a cohort study with a longitudinal design are required to confirm our conclusion and investigate the causal relationship.

## Conclusions

In summary, DR was positively associated with biscuit consumption. For male, non-Hispanic, or overweight individuals, the risk of DR significantly increased with the frequency of biscuit consumption. Our findings might provide beneficial dietary guidance for patients at risk of DR.

## Supplementary Information

**Additional file 1: Table S1.** Association of biscuit consumption with DR incidence in subgroups of confounders

## Data Availability

The datasets generated and analyzed during the current study are available in the National Health and Nutrition Examination Survey (NHANES) repository, https://wwwn.cdc.gov/nchs/nhanes/.

## Abbreviations

BMI: Body mass index
BP: Blood pressure
CI: Confidence interval
CVD: Cardiovascular disease
DM: Diabetic mellitus
DR: Diabetic retinopathy
FFQ: Food frequency questionnaire
HbA1c: Hemoglobin A1c
NHANES: National Health and Nutrition Examination Survey
PIR: Poverty income ratio
TFA: Trans fatty acids
