# Supplementary material for: Biscuit consumption and diabetic retinopathy incidence in adults in the United States
Source: Diabetol Metab Syndr. 2022 Jul 6;14:94. doi: 10.1186/s13098-022-00860-7 (PMC9258145; doi:10.1186/s13098-022-00860-7)
Supplement: Supplementary file 1 — Additional file 1: Table S1. Association of biscuit consumption with DR incidence in subgroups of confounders [file 13098_2022_860_MOESM1_ESM.docx]

**Supplementary Table 1.** **Association of biscuit consumption with DR incidence in subgroups of confounders**

|  | **OR (95% CI) *P*-value** | | | | ***P*_trend_** | ***P*_interaction_** |
| --- | --- | --- | --- | --- | --- | --- |
|  | **Never ate** | **1–11 times per year** | **1–3 times per month** | **≥1 times per week** |  |  |
| **Sex** |  |  |  |  |  |  |
| Male | Reference | 1.388 (0.678, 2.845) 0.370 | 1.702 (0.788, 3.677) 0.176 | 2.434 (1.066, 5.561) **0.035** | **0.021** | 0.597 |
| Female | Reference | 2.957 (1.026, 8.520) 0.045 | 2.029 (0.658, 6.254) 0.218 | 2.577 (0.799, 8.312) 0.113 | 0.585 |  |
| **Age (years)** |  |  |  |  |  |  |
| <60 | Reference | 3.884 (0.934, 16.143) 0.062 | 3.883 (0.898, 16.780) 0.069 | 5.225 (1.125, 24.279) **0.035** | 0.125 | 0.760 |
| ≥60 | Reference | 1.471 (0.750, 2.884) 0.261 | 1.412 (0.672, 2.964) 0.362 | 1.930 (0.885, 4.207) 0.098 | 0.141 |  |
| **Ethnicity** |  |  |  |  |  |  |
| Hispanic | Reference | 1.851 (0.534, 6.410) 0.331 | 0.825 (0.194, 3.501) 0.794 | 1.382 (0.299, 6.398) 0.679 | 0.673 | 0.529 |
| Non-Hispanic | Reference | 1.811 (0.923, 3.555) 0.084 | 2.209 (1.084, 4.501) **0.029** | 2.689 (1.262, 5.728) **0.010** | **0.009** |  |
| Other ethnicities | Reference | ɸ | ɸ | ɸ | ɸ |  |
| **Education level** |  |  |  |  |  |  |
| High school grade or below | Reference | 1.646 (0.812, 3.335) 0.167 | 1.188 (0.544, 2.595) 0.666 | 2.012 (0.902, 4.486) 0.087 | 0.307 | 0.366 |
| College or above | Reference | 2.164 (0.761, 6.152) 0.148 | 3.331 (1.113, 9.971) **0.031** | 3.008 (0.900, 10.051) 0.074 | **0.036** |  |
| **Marital status** |  |  |  |  |  |  |
| Partnered | Reference | 2.800 (1.214, 6.457) **0.016** | 2.791 (1.160, 6.716) **0.022** | 3.752 (1.497, 9.401) **0.005** | **0.021** | 0.478 |
| Single | Reference | 1.152 (0.485, 2.739) 0.749 | 1.218 (0.463, 3.206) 0.689 | 1.564 (0.560, 4.367) 0.394 | 0.389 |  |
| **Family PIR** |  |  |  |  |  |  |
| <1.3 | Reference | 1.246 (0.433, 3.580) 0.684 | 0.895 (0.275, 2.908) 0.853 | 1.947 (0.582, 6.511) 0.280 | 0.451 | 0.694 |
| 1.3–3.5 | Reference | 1.754 (0.773, 3.979) 0.179 | 1.648 (0.669, 4.058) 0.277 | 2.014 (0.784, 5.170) 0.146 | 0.272 |  |
| ≥3.5 | Reference | 2.796 (0.611, 12.789) 0.185 | 4.854 (1.011, 23.297) **0.048** | 5.480 (1.017, 29.546) **0.048** | **0.014** |  |
| **Smoking consumption habit** |  |  |  |  |  |  |
| ≥100 cigarettes in life | Reference | 1.432 (0.682, 3.007) 0.342 | 1.756 (0.787, 3.916) 0.169 | 1.590 (0.656, 3.854) 0.305 | 0.254 | 0.205 |
| <100 cigarettes in life | Reference | 2.468 (0.913, 6.669) 0.075 | 1.885 (0.658, 5.401) 0.238 | 3.954 (1.346, 11.617) **0.012** | 0.058 |  |
| **Alcohol consumption habit** |  |  |  |  |  |  |
| ≥12 alcohol drinks/year | Reference | 1.588 (0.772, 3.266) 0.209 | 1.881 (0.869, 4.071) 0.109 | 2.215 (0.939, 5.226) 0.069 | 0.061 | 0.487 |
| <12 alcohol drinks/year | Reference | 2.236 (0.804, 6.217) 0.123 | 1.559 (0.525, 4.629) 0.424 | 2.343 (0.773, 7.097) 0.132 | 0.473 |  |
| **Blood glucose level (mmol/l)** |  |  |  |  |  |  |
| <6.9 | Reference | 1.259 (0.517, 3.069) 0.612 | 1.355 (0.524, 3.505) 0.531 | 1.402 (0.495, 3.969) 0.524 | 0.536 | 0.862 |
| ≥6.9 | Reference | 1.160 (0.258, 5.221) 0.846 | 2.009 (0.382, 10.558) 0.410 | 1.161 (0.136, 9.902) 0.892 | 0.539 |  |
| **Hemoglobin A1c (HbA1c, %)** |  |  |  |  |  |  |
| < 6.5 | Reference | 1.510 (0.782, 2.916) 0.219 | 1.618 (0.801, 3.271) 0.180 | 2.307 (1.098, 4.845) **0.027** | **0.031** | 0.461 |
| ≥6.5 | Reference | 4.123 (1.229, 13.831) **0.022** | 3.807 (1.018, 14.240) **0.047** | 3.575 (0.856, 14.923) 0.081 | 0.286 |  |
| **Diagnosis of diabetes** |  |  |  |  |  |  |
| Yes | Reference | 3.921 (1.037, 14.830) 0.044 | 4.854 (1.204, 19.579) **0.026** | 4.277 (0.949, 19.271) 0.059 | 0.147 | 0.464 |
| No | Reference | 1.326 (0.680, 2.588) 0.408 | 1.295 (0.626, 2.681) 0.486 | 1.744 (0.803, 3.785) 0.160 | 0.218 |  |
| Borderline | Reference | ɸ | ɸ | ɸ | ɸ |  |
| **Insulin use** |  |  |  |  |  |  |
| Yes | Reference | 0.128 (0.000, 34.225) 0.471 | 122.801 (0.167, 90438.518) 0.153 | 0.084 (0.000, 49.975) 0.448 | 0.501 | 0.089 |
| No | Reference | 1.659 (0.908, 3.031) 0.0999 | 1.520 (0.791, 2.921) 0.209 | 2.128 (1.063, 4.258) **0.033** | 0.095 |  |
| **Systolic blood pressure (mmHg)** |  |  |  |  |  |  |
| <120 | Reference | 4.697 (0.909, 24.284) 0.065 | 3.126 (0.562, 17.375) 0.193 | 5.159 (0.895, 29.733) 0.066 | 0.317 | 0.690 |
| 120–140 | Reference | 1.335 (0.546, 3.261) 0.526 | 1.882 (0.721, 4.912) 0.197 | 1.649 (0.557, 4.877) 0.366 | 0.205 |  |
| ≥140 | Reference | 1.740 (0.665, 4.553) 0.259 | 1.788 (0.632, 5.053) 0.273 | 2.677 (0.916, 7.820) 0.072 | 0.093 |  |
| **Diastolic blood pressure (mmHg)** |  |  |  |  |  |  |
| <80 | Reference | 1.616 (0.848, 3.080) 0.145 | 1.572 (0.780, 3.169) 0.206 | 2.054 (0.979, 4.310) 0.057 | 0.114 | 0.308 |
| 80–90 | Reference | 0.947 (0.179, 5.028) 0.950 | 0.976 (0.166, 5.738) 0.979 | 0.892 (0.119, 6.654) 0.911 | 0.941 |  |
| ≥90 | Reference | ɸ | ɸ | ɸ | ɸ |  |
| **BMI (kg/m^2^)** |  |  |  |  |  |  |
| <25 | Reference | 0.937 (0.332, 2.644) 0.902 | 0.943 (0.291, 3.060) 0.923 | 0.550 (0.141, 2.151) 0.391 | 0.421 | 0.136 |
| ≥25 | Reference | 2.759 (1.295, 5.879) **0.009** | 2.768 (1.244, 6.159) **0.013** | 4.376 (1.898, 10.086) **0.0005** | **0.002** |  |

ɸ, the model failed because of the small sample size. OR, odds ratio; CI, confidence interval; BMI, body mass index.
